# Mortality in the 2011 Tsunami in Japan

**DOI:** 10.2188/jea.JE20120114

**Published:** 2013-01-05

**Authors:** Shinji Nakahara, Masao Ichikawa

**Affiliations:** 1 Department of Epidemiology and Health Promotion, St. Mariana University, Kawasaki, Kanagawa, Japan; 1 聖マリアンナ医科大学医学部; 2 Faculty of Medicine, University of Tsukuba, Tsukuba, Ibaraki, Japan; 2 筑波大学医学医療系

**Keywords:** disaster planning, epidemiology, regional differences, school safety, tsunamis

## Abstract

**Introduction:**

On 11 March 2011, a magnitude 9.0 earthquake caused a huge tsunami that struck Northeast Japan, resulting in nearly 20 000 deaths. We investigated mortality patterns by age, sex, and region in the 3 most severely affected prefectures.

**Methods:**

Using police data on earthquake victims in Iwate, Miyagi, and Fukushima prefectures, mortality rates by sex, age group, and region were calculated, and regional variability in mortality rates across age groups was compared using rate ratios (RRs), with the rates in Iwate as the reference.

**Results:**

In all regions, age-specific mortality showed a tendency to increase with age; there were no sex differences. Among residents of Iwate, mortality was markedly lower among school-aged children as compared with other age groups. In northern Miyagi and the southern part of the study area, RRs were higher among school-aged children than among other age groups.

**Conclusions:**

The present study could not address the reasons for the observed mortality patterns and regional differences. To improve preparedness policies, future research should investigate the reasons for regional differences.

## Abstract (ja)

**【背景】:**

2011年3月11日に発生したマグニチュード9.0の東北地方太平洋沖地震は、東北地方太平洋沿岸に大津波による壊滅的被害をもたらし、20,000人近い死者行方不明者が発生した。本研究では最も大きな被害を受けた岩手県、宮城県、福島県における年齢、性、地域別の死亡パターンを分析した。

**【方法】:**

警察発表の岩手県、宮城県、福島県における地震による死亡者データを用いて、性別、年齢層別、地域別の死亡率を算出した。年齢層別死亡率の地域差は岩手県を参照カテゴリとする死亡率比により比較した。

**【結果】:**

すべての地域で、年齢別死亡率は年齢とともに上昇する傾向があったが、性差は見られなかった。岩手県では、学齢期の子供が他の年齢層に比べて著しく低い死亡率を示した。宮城県北部、南部地域（宮城県南部と福島県）で、学齢期の子供は他の年齢に比べて高い死亡率比を示した。

**【結論】:**

本研究では死亡パターンの地域差の原因を明らかにすることはできなかったが、災害による被害を最小限に抑えるための防災対策を改善するためには、これらの地域差の原因を明らかにしていく必要がある。

## INTRODUCTION

On 11 March 2011, a magnitude 9.0 earthquake caused a huge tsunami that resulted in catastrophic damage to Northeast Japan and nearly 20 000 deaths.^1^^–^^4^ The tsunami overrode coastal defenses and engulfed towns throughout the affected areas; there were particularly high numbers of human casualties in the coastal areas of Iwate, Miyagi, and Fukushima prefectures, and more than 99% of all deaths were in these areas.^2^ Most deaths were caused by the tsunami rather than by building collapse.^1^

Improvement of disaster mitigation and management plans requires an understanding of the patterns of human casualties.^5^ Epidemiologic studies describing mortality patterns in relation to individual and community-level characteristics would provide useful information on vulnerable groups, which can be a starting point in developing effective protection plans against large natural disasters.

Epidemiologic studies of casualty patterns in tsunamis are relatively rare because tsunamis are less frequent than other disasters such as earthquakes and floods. During the past decade, the casualty patterns of the Indian Ocean tsunami in 2004 were reported.^6^^–^^9^ The objective of this study was to describe the mortality patterns of the Japanese tsunami on 11 March 2011 by sex, age group, and region in the 3 most severely damaged prefectures.

## METHODS

Earthquake-related mortality data (as of 11 November 2011) were obtained from the National Police Agency (NPA), and population data were obtained from the 2010 census. The NPA data included the age, sex, and address of victims, but not cause of death. Since it was not possible to distinguish tsunami-related deaths from crash deaths, and because 92% of deaths were caused by drowning,^1^ the analysis included all cases.

**Figure 1.**
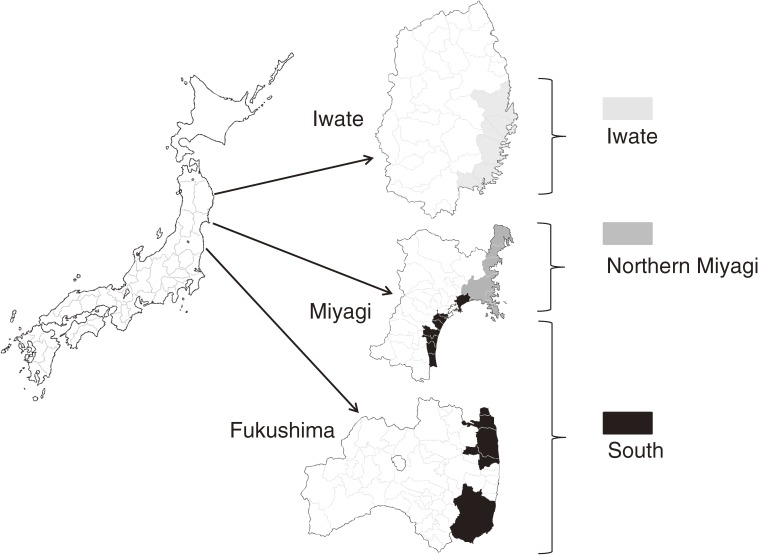
Map of the study area. Municipalities severely damaged by the tsunami were included in the analysis and were classified into 3 areas according to their geographic characteristics. Iwate includes Miyako, Ofunato, Rikuzentakata, Kamaishi, Otsuchi, and Yamada. Northern Miyagi includes Kesennuma, Minamisanriku, Ishinomaki, and Onagawa. The South includes Higashimatsushima, Tagajo, Sendai (Miyagino and Wakabayashi), Natori, Iwanuma, Watari, and Yamamoto in Miyagi prefecture, and Shinchi, Soma, Minamisoma, Namie, and Iwaki in Fukushima prefecture.

In an attempt to exclude trauma deaths, the analysis included municipalities with 100 or more deaths (based on the assumption that municipalities affected by the earthquake alone would have few deaths; Figure 1). Due to the influence of geographic and social traits on patterns of inundation and casualties, we compared northern Miyagi and Iwate. These regions share common traits, namely, a deeply indented coastline with narrow flatlands bordered by sea and mountains (which increases vulnerability to tsunami damage) and citizens who tend to work within their municipalities.^10^ Southern Miyagi and Fukushima (hereafter; the South) were combined for an additional comparison with Iwate, due to their common traits, ie, a smooth coastline with broader flatlands and citizens who tend to work outside their municipalities.

Mortality rates were calculated by sex, age group, and region; age-specific rate ratios (RRs) and 95% CIs were calculated using the rates in Iwate as reference (because Iwate had a unique child mortality pattern). CIs were calculated using the method described by Morris and Gardner.^11^ Victim data were entered into the police database by agreement with their families. The institutional review board of St. Mariana University approved the study protocol and waived the requirement for informed consent.

We conducted a sensitivity analysis of the potential effect on regional differences of a major loss of life in 1 school in northern Miyagi—74 children died or were missing because of the late evacuation of the school, a death toll that was much larger than that of other schools in the affected areas.^12^ The RR for children aged 5 to 14 years in northern Miyagi was recalculated after removing the data from this school.

## RESULTS

**Figure 2.**
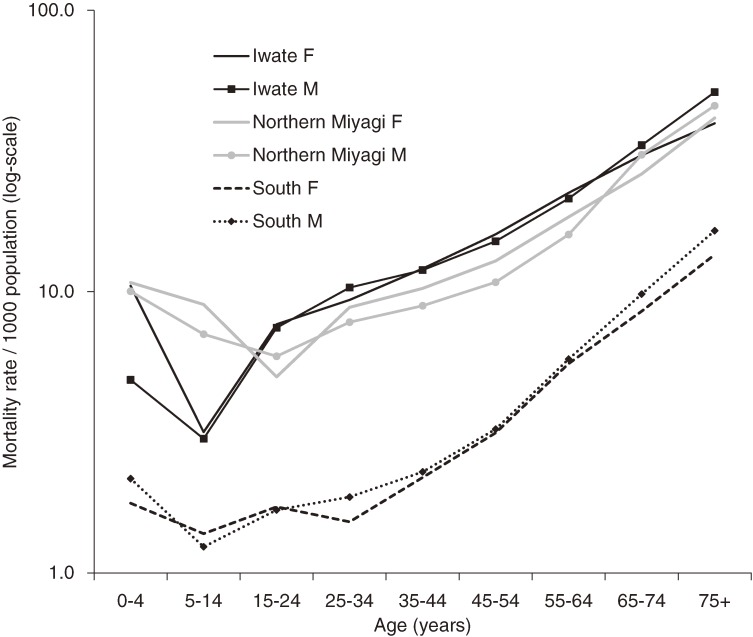
Mortality rates by sex, age, and region. F, female; M, male.

Of the 15 770 deaths confirmed in the 3 prefectures, 14 931 were included in the NPA data and 14 220 were in the selected municipalities; the analyses included the 13 932 deaths with complete data. Children younger than 15 years and adults 65 years or older accounted for 4.3% and 56.7%, respectively, of the total deaths (Table). In all areas, there was no sex difference in mortality; age-specific mortality showed a tendency to increase with age (Figure 2). In comparisons among age groups within a region, children aged 5 to 14 years had the lowest mortality rates in Iwate and the South, and people aged 15 to 24 years had the lowest rate in northern Miyagi. Children aged 0 to 4 years had higher mortality rates than those aged 5 to 14 years in all areas.

**Table.** Number of deaths by region, sex, and age group

Age (years)	Iwate				Northern Miyagi				South			
		
	Female		Male		Female		Male		Female		Male	
					
	*n*	%	*n*	%	*n*	%	*n*	%	*n*	%	*n*	%
0–4	33	1.5	16	0.8	47	1.8	46	2.2	41	1.6	52	2.1
5–14	26	1.2	26	1.4	102	3.9	84	4.0	68	2.6	64	2.6
15–24	53	2.4	53	2.8	53	2.0	66	3.1	90	3.4	91	3.7
25–34	78	3.5	87	4.5	111	4.3	102	4.8	105	4.0	130	5.3
35–44	133	6.0	136	7.1	156	6.0	138	6.5	160	6.1	174	7.2
45–54	194	8.7	184	9.6	216	8.3	180	8.5	210	8.0	219	9.0
55–64	369	16.5	337	17.6	389	15.0	333	15.7	436	16.5	451	18.5
65–74	513	23.0	452	23.6	510	19.7	517	24.4	529	20.0	535	22.0
75+	834	37.3	626	32.7	1005	38.8	654	30.8	1000	37.9	717	29.5

Total	2233	100.0	1917	100.0	2589	100.0	2120	100.0	2639	100.0	2433	100.0

**Figure 3.**
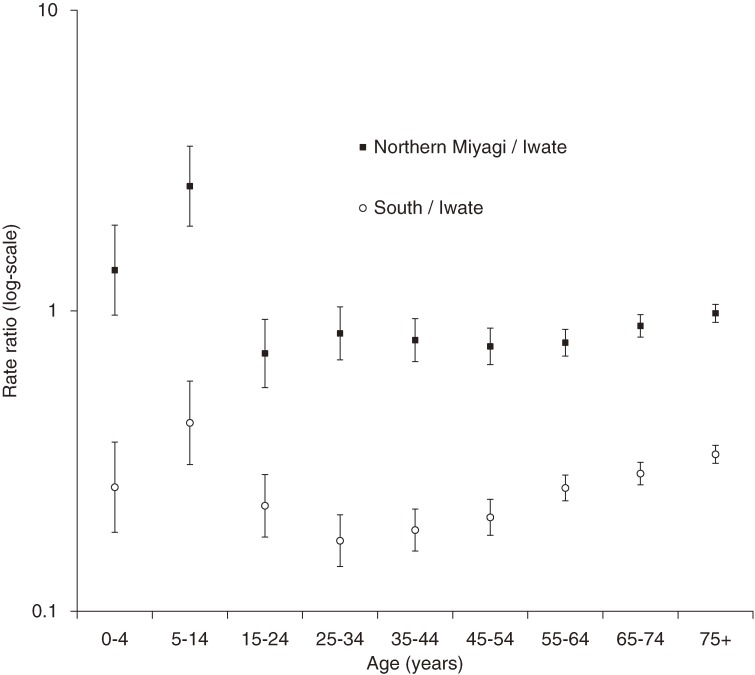
Mortality rate ratios and 95% CIs, with mortality rates in Iwate as the reference.

In northern Miyagi, school-aged children had a higher RR (2.60; 95% CI: 1.91–3.53) than adults (0.72–0.98) (Figure 3). The South, which had lower overall RRs than northern Miyagi, also showed a similar difference between school-aged children and adults. However, variability among adults was greater. In the sensitivity analysis, school-aged children in northern Miyagi had a RR of 1.56 (95% CI: 1.13–2.17) after excluding the data from the 1 school with a particularly high number of deaths.

## DISCUSSION

This study revealed mortality patterns of the 11 March tsunami by sex and age group. These patterns differed from those observed in Indonesia and Sri Lanka during the Indian Ocean tsunami in 2004. Whereas studies in Indonesia and Sri Lanka reported higher mortality rates among children, elderly adults, and women,^6^^–^^9^ the present study found lower mortality rates among children, increasing rates with age, and no sex differences. In addition, we noted regional variation in child mortality, with a pronounced dip in the mortality of school-aged children among victims in Iwate only.

The timing of the tsunami might have influenced age–sex mortality patterns. As with earthquakes,^13^ the risk of being caught in a tsunami may be related to time of day, which influences the whereabouts of people. The Indian Ocean tsunami hit rural communities on Sunday morning, when children and women were at home but men were working away from home (eg, engaged in offshore fishing).^7^ The 11 March tsunami hit communities in the afternoon on a weekday, when children were attending school, kindergarten, and out-of-home daycare. Schools might have provided better protection than households. Preschool children had higher mortality rates than school-aged children, which might have reflected the fact that preschool children do not necessarily attend a kindergarten or daycare, and younger children in particular might have been cared for at home.

The presence of a better tsunami warning system in Japan may explain the lack of a sex difference in mortality, although the system did not work as expected in some areas. Most people at home (women and elderly adults) evacuated immediately after the earthquake, following evacuation calls or even before such calls.^14^ Higher mortality among elderly adults was seen in all areas and suggests that they could not evacuate promptly or could not withstand the force of the tsunami, as discussed in previous reports.^7^ The lower overall mortality rates in the South may be due to the greater expanse of flatlands and the larger number of people living inland, and thus the smaller proportion of people inundated, in contrast to the situation in Iwate and northern Miyagi, where most of the population live in narrow coastal strips.

Regional differences in child mortality rates might have resulted from different levels of preparedness at schools, given that the earthquake occurred during school hours in most schools in the affected areas: 72% of kindergartens, 91% of primary schools, and 59% of junior high schools were affected during school hours.^15^ Only 50% of the schools affected by the tsunami had prepared an evacuation plan for children,^15^ and actual evacuation practices seemed to differ across schools, although it was standard practice to return children to their parents immediately after the disaster.^16^ Anecdotal evidence indicates that immediate evacuation to safe sites, without returning children home, saved children, as shown in a school in Iwate,^17^ whereas nearly 80% of deaths among school-aged children occurred outside school (eg, they left school with parents after the earthquake, had already returned home before the earthquake, or were absent from school).^18^

The limitations of this study include the fact that the analyzed data did not have more detailed information on causes, locations, and circumstances of deaths. Therefore, we could not investigate in detail the reasons for the observed mortality patterns or explain differences from previous reports on the Indian Ocean tsunami. However, we have presented possible explanations for the findings, as discussed above, which provide a basis for further research on factors that determine vulnerability and mortality risk. Such investigations will provide useful information for future disaster preparedness.^14^

## ONLINE ONLY MATERIALS

The Japanese-language abstract for articles can be accessed by clicking on the tab labeled Supplementary materials at the journal website http://dx.doi.org/10.2188/jea.JE20120114.

Abstract in Japanese.
