# Supplementary material for: Mortality in the 2011 Tsunami in Japan
Source: J Epidemiol. 2013 Jan 5;23(1):70–3. doi: 10.2188/jea.JE20120114 (PMC3700238; doi:10.2188/jea.JE20120114)
Supplement: Abstract in Japanese. [file je-23-070-s001.pdf]

2011年東北地方太平洋沖地震に伴う津波による東北三県の死亡パターン

中原慎二[1]、市川政雄[2]

[1]聖マリアンナ医科大学医学部、[2]筑波大学医学医療系

【背景】2011年3月11日に発生したマグニチュード9.0の東北地方太平洋沖地震は、東北地方太平洋沿岸に大津波による壊滅的被害をもたらし、20,000 人近い死者行方不明者が発生した。本研究では最も大きな被害を受けた岩手県、宮城県、福島県における年齢、性、地域別の死亡パターンを分析した。

【方法】警察発表の岩手県、宮城県、福島県における地震による死亡者データを用いて、性別、年齢層別、地域別の死亡率を算出した。年齢層別死亡率の地域差は岩手県を参照カテゴリとする死亡率比により比較した。

【結果】すべての地域で、年齢別死亡率は年齢とともに上昇する傾向があったが、性差は見られなかった。岩手県では、学齢期の子供が他の年齢層に比べて著しく低い死亡率を示した。宮城県北部、南部地域（宮城県南部と福島県）で、学齢期の子供は他の年齢に比べて高い死亡率比を示した。

【結論】本研究では死亡パターンの地域差の原因を明らかにすることはできなかったが、災害による被害を最小限に抑えるための防災対策を改善するためには、これらの地域差の原因を明らかにしていく必要がある。

キーワード：防災計画、災害疫学、地域差、学校安全、津波
